# The influence of primary care physicians’ mental health knowledge, attitudes and self-efficacy on referrals to specialised services: findings from a longitudinal pilot trial

**DOI:** 10.1192/bjo.2020.115

**Published:** 2020-10-30

**Authors:** Jessica Spagnolo, Helen-Maria Vasiliadis, Djamal Berbiche, François Champagne, Nicole Leduc, Wahid Melki, Khalid Saeed, Fatma Charfi

**Affiliations:** School of Public Health, University of Montreal; Department of Community Health Sciences, University of Sherbrooke; and Charles-Le-Moyne - Saguenay-Lac-St-Jean Research Centre on Health Innovations, University of Sherbrooke - Longueuil Campus, Quebec, Canada; Department of Community Health Sciences, University of Sherbrooke; and Charles-Le-Moyne - Saguenay-Lac-St-Jean Research Centre on Health Innovations, University of Sherbrooke - Longueuil Campus, Quebec, Canada; Department of Community Health Sciences, University of Sherbrooke; and Charles-Le-Moyne - Saguenay-Lac-St-Jean Research Centre on Health Innovations, University of Sherbrooke - Longueuil Campus, Quebec, Canada; School of Public Health, University of Montreal, Quebec, Canada; School of Public Health, University of Montreal, Quebec, Canada; Department of Psychiatry D, Razi Hospital; Faculty of Medicine of Tunis, University of Tunis El-Manar, Tunisia; and Technical Committee for Mental Health Promotion in Ministry of Health, Tunis, Tunisia; Mental Health and Substance Abuse Unit, Department of Non-Communicable Diseases and Mental Health, World Health Organization Regional Office for the Eastern Mediterranean, Egypt; Department of Child Psychiatry, Mongi Slim Hospital; and Faculty of Medicine of Tunis, University of Tunis El-Manar, Tunisia

**Keywords:** Primary care, Tunisia, education and training, outcome studies

## Abstract

**Background:**

Training based on the Mental Health Gap Action Programme (mhGAP) is being increasingly adopted by countries to enhance non-specialists’ mental health capacities. However, the influence of these enhanced capacities on referral rates to specialised mental health services remains unknown.

**Aims:**

We rely on findings from a longitudinal pilot trial to assess the influence of mental health knowledge, attitudes and self-efficacy on self-reported referrals from primary to specialised mental health services before, immediately after and 18 months after primary care physicians (PCPs) participated in an mhGAP-based training in the Greater Tunis area of Tunisia.

**Method:**

Participants included PCPs who completed questionnaires before (*n* = 112), immediately after (*n* = 88) and 18 months after (*n* = 59) training. Multivariable analyses with linear mixed models accounting for the correlation among participants were performed with the SAS version 9.4 PROC MIXED procedure. The significance level was α < 0.05.

**Results:**

Data show a significant interaction between time and mental health attitudes on referrals to specialised mental health services per week. Higher scores on the attitude scale were associated with more referrals to specialised services before and 18 months after training, compared with immediately after training.

**Conclusion:**

Findings indicate that, in parallel to mental health training, considering structural/organisational supports to bring about a sustainable change in the influence of PCPs’ mental health attitudes on referrals is important. Our results will inform the scale-up of an initiative to further integrate mental health into primary care settings across Tunisia, and potentially other countries with similar profiles interested in further developing task-sharing initiatives.

## The Mental Health Gap Action Programme training

The Mental Health Gap Action Programme (mhGAP) was developed by the World Health Organization (WHO) to bridge the mental health treatment gap (i.e. the gap between the need for mental health services and their delivery^1,2^) by developing technical guidance and tools to support decision makers, programme managers and healthcare organisations.^3^ The programme was specifically designed for low- and middle-income countries (LMICs) where the treatment gap is estimated at 76–85%,^2^ given financial, infrastructure and human resource challenges that influence access to timely mental healthcare.^4^ One of the mhGAP tools is the Intervention Guide (mhGAP-IG), evidence-based guidance to train and support non-specialists in better detecting, treating and managing what the WHO considers priority mental, neurological and substance use disorders (MNS).^3^

In 2016, a mental health training programme based on the mhGAP-IG was offered to primary care physicians (PCPs) working in the Greater Tunis area of Tunisia, a lower-middle-income country in North Africa. The implementation and evaluation of the training programme based on the mhGAP-IG (version 1.0)^5^ in Tunisia was a collaborative effort between members of the Ministry of Health in Tunisia (particularly the President of the Committee for Mental Health Promotion and the Coordinator of the Technical Committee for Suicide Prevention), the School of Public Health at Université de Montréal (Canada) and the WHO office in Tunisia.^6^

## Factors influencing referral rates

Although the mhGAP-based training has shown effectiveness in improving non-specialists’ mental health capacities to detect and manage MNS disorders,^7^ to our knowledge, there are no studies that assess the influence of mental health capacity (i.e. mental health knowledge, attitudes, confidence in capabilities to detect and manage MNS disorders) acquired through mhGAP-based training on non-specialists’ referral rates to specialised mental health services.^7^

A range of factors could be associated with non-specialists’ referral rates to specialised mental healthcare. For example, studies show that confidence in capacities to treat mental health conditions^8–10^ and knowledge about these conditions,^11^ as well as attitudes toward specific mental health problems (i.e. what non-specialists may consider to be complex conditions like psychosis and symptoms related to suicidality) or types of patients (i.e. those non-responsive to prescribed medications) are strong predictors of referrals from primary to specialised mental healthcare.^9^

## Study aim

The present study aims to improve understanding of the influence of mental health knowledge, attitudes and self-efficacy on self-reported referrals by PCPs to specialised mental health services at three time points: before, immediately after and 18 months after PCPs’ participation in the mhGAP-based mental health training programme offered in the Greater Tunis area of Tunisia. Understanding how these capacities influence non-specialists’ referrals to specialised mental health services at these three time points will fill a gap in the literature. Findings will provide evidence that may help improve the mhGAP-based training programme design to further foster capacity-building in non-specialised settings; inform the scale-up of a task-sharing programme in Tunisia, centred on further integrating mental health in primary care settings by increasing non-specialists’ involvement in mental healthcare delivery and support by specialists; and be relevant to other countries with similar primary care realities and interested in further integrating mental health in primary care settings through, for example, task-sharing initiatives.

## Method

### Study design

**Fig. 1:**
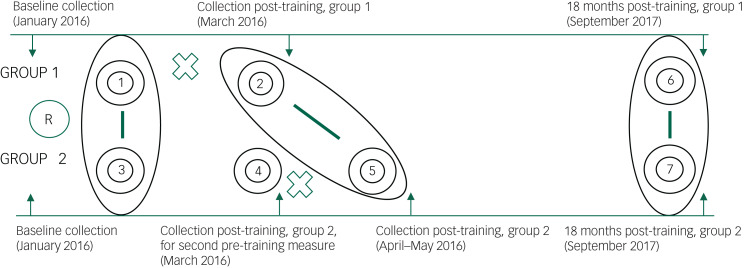
The design of the pilot trial in which this study is inscribed. Our study is inscribed within a pilot trial that aimed to assess the mental health capacities of primary care physicians (PCPs) after their participation in Mental Health Gap Action Programme (mhGAP)-based training. For the purposes of this study, we rely on a repeated measures design (as shown in Fig. 1) to improve understanding of the influence of mental health knowledge, attitudes and self-efficacy on self-reported referrals to specialised mental health services at three time periods: pre-training (circles 1 and 3), immediately post-training (circles 2 and 5) and 18 months post-training (circles 6 and 7). X represents the implementation of the training. R represents when PCPs were randomized to either group 1 or group 2. The circled areas depict the pooling of group 1 and group 2 over three time periods. This study relies on the secondary data analysis of data collected during the pilot evaluation of the mhGAP-based training in the Greater Tunis area of Tunisia. The pilot study design in which this study is inscribed has been described in detail elsewhere.^6^

This study relies on data collected for the pilot evaluation of the mhGAP-based training in the Greater Tunis area of Tunisia. The pilot study design has been described in detail elsewhere.^6^ In brief, we conducted a pilot trial between January 2016 and September 2017 to assess the effect of the training on PCPs’ mental health knowledge, attitudes, self-efficacy and self-reported practice, using several designs. First, a pre-test–post-test control group design^12^ was employed to assess the training's short-term effects (circles 1–4, Fig. 1). In this design, PCPs were randomly assigned to two groups: the intervention group (group 1) and the control group (group 2). Group 1 received the mhGAP-based training between February and March 2016 (circles 1 and 2, Fig. 1), and group 2 received the training between March and April 2016 (circles 4 and 5, Fig. 1). Second, a repeated measures design was employed to assess the training's long-term effects.

For this study, we employed a repeated measures design to further assess data from our pilot evaluation of the mhGAP-based training.^6^ Specifically, we assessed the influence of mental health knowledge, attitudes and self-efficacy on self-reported referrals to specialised mental health services over three time periods, as illustrated in Fig. 1: before the training (circles 1 and 3), immediately after the training of both groups (circles 2 and 5) and 18 months after the training of both groups (circles 6 and 7).

### Study setting

The evaluation of the mhGAP-based training on PCPs’ mental health capacities was conducted in the Greater Tunis area of Tunisia, located in the north of the country.^6^ This area includes four governorates: Ariana, Ben Arous, Manouba and Tunis. The Greater Tunis area was chosen as the pilot setting for the first implementation of the mhGAP-based training because it is diverse: it includes governorates that are rural, urban, semi-rural and semi-urban, which reflects Tunisia as a whole.

The authors assert that all procedures contributing to this work comply with the ethical standards of the relevant national and institutional committees on human experimentation and with the Helsinki Declaration of 1975, as revised in 2008. All procedures involving human subjects/patients were approved by the ethics committees of Université de Montréal (Québec, Canada) (approval number #15-117-CERES-D) and Hôpital Razi (Tunisia).

During the recruitment phase, PCPs were presented the training programme and the study. They were informed that the training and the study were voluntary, and that their decisions related to participation in the training and study would not have any adverse consequences on their employment. Informed consent was obtained from all study participants.

### Participants

Participant recruitment for the pilot trial in which this study is inscribed has been described in detail elsewhere.^6^ We compiled the names of 345 PCPs who were registered with the PCPs’ professional order in Tunisia, worked in the public and primary care sectors and previously attended continuing medical education training. Continuing medical education is recommended and encouraged in Tunisia for advancing PCPs’ careers. Of the 345 PCPs, the majority (91.30%) worked in primary care in the Greater Tunis area and had 5 or more years of clinical experience, and so were eligible for the study. A total of 132 PCPs (41.90%) agreed to participate in the pilot trial. In January 2016, the first author contacted the 132 PCPs who agreed to participate in the trial, to further explain the study and to obtain consent. A total of 112 (*n* = 112) PCPs completed baseline questionnaires and were thus included in the trial.

Forty-five PCPs from group 1 and 43 PCPs from group 2 completed the training programme and agreed to complete questionnaires. This resulted in a pooled total of 88 PCPs included in the analyses immediately after the training. Thirty-two PCPs from group 1 and 27 PCPs from group 2 agreed to complete questionnaires at 18 months (September 2017) post-training. This resulted in a pooled total of 59 PCPs included in long-term analysis.

### Intervention

The mhGAP-based training (version 1.0)^5^ was offered to PCPs in the Greater Tunis area between February and May 2016. The programme's adaptation to the local context has been described elsewhere.^13^ Members of the Ministry of Health in Tunisia (W.M., F. Charfi) chose specific training modules considered important to Tunisia: general principles of care, depression, psychosis, suicide/self-harm and problems related to substance use. These modules were adapted to meet the Greater Tunis area's primary care realities, with the help of W.M., F. Charfi, three Tunisian psychiatrists (‘trainers’) and seven PCPs responsible for continuing medical education in the Greater Tunis area who had the role of supporting trainees during and post-training (‘tutors’). The training lasted 6 weeks for a total of 19 h, with sessions being offered once a week. The first five sessions comprised general lectures, role-plays and group discussions (17 h). The last session was a 2-h support session offered by the trainers consisting of further role-plays to help PCPs with challenging mental health cases.

### Measures

Variables for the pilot trial^6^ were chosen according to Kirkpatrick's conceptual model,^14^ often used for training programme evaluations.^15^

### Independent variables

Mental health knowledge was assessed using the questionnaire developed by the WHO to accompany the mhGAP training programme. The questionnaire contained 16 questions on the modules selected for training. Correct answers were scored as 1 and incorrect answers were scored as 0. A participant's score is the sum of correct answers for individual items. Overall knowledge scores were converted to a score ranging from 0 to 10, where a higher score indicates more knowledge. This questionnaire reported a good degree of reliability in our sample (the average measure Intraclass Correlation Coefficient (ICC) was 0.708, with a 95% confidence interval of 0.478–0.837).^16^ These psychometric analyses have been reported elsewhere.^16^

The Mental Illness: Clinicians’ Attitudes (MICA) Scale (version 4.0) was used to measure attitudes toward mental illness and the field of mental health.^17–18^ The scale's Cronbach's alpha, tested on our sample, was found to be poor (0.521). To increase the scale's internal consistency, we kept 11 items (i.e. questions 1, 2, 4, 5, 7, 10, 12, 13, 14, 15 and 16) from the MICA-4 (version 4.0), which resulted in an increase in the total Cronbach's alpha (0.608). Cronbach's alpha is a function of scale length, and increased in our case by removing five items from the original scale. Hence, the new Cronbach's alpha was sufficient.^16^ This procedure has been explained in detail elsewhere.^16^ For statements 10, 12 and 16, items were scored on a scale of 1–6, from ‘strongly agree’ to ‘agree’, ‘somewhat agree’, ‘somewhat disagree’, ‘disagree’ and ‘strongly disagree’. All other items were reverse scored. Scores on individual items were summed to obtain each participant's overall score within a range of 11–66 points. A higher global score indicates a more negative perception about MNS disorders and mental healthcare delivery. This modified questionnaire reported a good degree of reliability in our sample (the average measure of the ICC was 0.704, with a 95% confidence interval of 0.468–0.835).^16^

The self-efficacy questionnaire was developed for the pilot trial and comprised 35 questions about PCPs’ judgement of their capability to detect, treat and manage mental health conditions included in the training programme. The scale's Cronbach's alpha, tested on our sample, was found to be satisfactory (0.937). Each statement was scored on a scale of 0–4, from ‘strongly agree’ to ‘somewhat agree’, ‘neutral’, ‘somewhat disagree’ and ‘strongly disagree’. A participant's overall score is the sum of answers for individual items. Overall scores were converted to a score ranging from 0 to 10, where a higher score indicates more self-efficacy. This questionnaire has shown a good degree of reliability in our sample (the average measure of the ICC was 0.781, with a 95% confidence interval of 0.606–0.878).^16^ These psychometric analyses have been reported elsewhere.^16^

### Dependent variable

Our variable of interest was PCPs’ self-reported referrals to specialised mental health services, which represents the average weekly percentage of mental health clientele that PCPs reported referring to specialised mental health services. Specifically, PCPs were asked the following question related to their self-reported referrals: among patients presenting with mental health problems, what percentage do you refer to specialised mental health services per week? The variable ‘self-reported referrals to specialised mental health services’ is evaluated on a score that ranges between 0 and 100%.

### Covariates

Baseline sociodemographic (age, gender) and practice characteristics, such as part-time or full-time work, average number of weekly work hours, average number of weekly hours dedicated to mental health, average number of weekly patient consultations, average number of weekly patient consultations specifically for mental health, average weekly percentage of consultations per type of mental health condition and previous mental health training, were examined as potential covariates.

### Data collection

All questionnaires were pre-tested^16^ and administered at four time points: at baseline, before randomisation (January 2016); following group 1's training (March 2016); following group 2's training (April and May 2016); and 18 months after the training (September 2017) (Fig. 1).

### Analyses

This study is a secondary analysis of data collected within the context of a pilot trial.^6^ To assess whether PCPs’ mental health knowledge, attitudes and self-efficacy predict PCPs’ self-reported referrals to specialised mental health services, we conducted multiple linear regression analyses. Multivariable analyses with linear mixed models, accounting for the correlation among participants, were performed with the SAS version 9.4 PROC MIXED procedure (platform X64_8PRO for Windows). One of the strengths of these models is that they consider an unequal number of measurements per participant (owing to attrition and/or non-response) for a given time period and even if time intervals are not constant. The level of significance was set at α < 0.05.

## Results

Table 1 summarises the sociodemographic and practice characteristics of PCPs who completed questionnaires before participation in the training (*n* = 112), after PCPs assigned to groups 1 and 2 participated in the training (*n* = 88) and 18 months after training (*n* = 59).

**Table 1:** Primary care physicians’ sociodemographic and practice characteristics at three data collection times

Sociodemographic characteristics	Time of data collection				
	Before (*n* = 112)	Immediately after (*n* = 88)	*P* value^a^	18 months after (*n* = 59)	*P* value^b^
Age, years, median (Q1, Q3)	49.00 (46.00, 53.00)	48.00 (45.00, 52.00)	0.209	48.00 (45.00, 52.00)	0.121
Gender, *n* (%)					
Women	90 (80.40)	73 (83.00)	0.244	50 (84.70)	0.523
Men	22 (19.60)	15 (17.00)		9 (15.30)	
Practice characteristics					
Governorate, *n* (%)					
Tunis	43 (38.40)	33 (37.50)	0.341	22 (37.30)	0.519
Manouba	21 (18.80)	16 (18.20)		10 (16.90)	
Ben Arous	20 (17.90)	14 (15.90)		9 (15.30)	
Ariana	28 (25.00)	25 (28.40)		18 (30.50)	
Work, *n* (%)					
Part time	28 (25.00)	19 (21.60)	0.110	10 (16.90)	0.131
Full time	84 (75.00)	69 (78.40)		49 (83.10)	
Hours of work per week, median (Q1, Q3)	36.00 (30.00, 36.00)	36.00 (34.00, 36.00)	0.693	36.00 (36.00, 36.00)	0.193
Average number of hours dedicated to mental healthcare per week, median (Q1, Q3)	3.60 (2.10, 6.00)^c^	3.60 (2.16, 7.20)	0.886	3.60 (1.08, 7.20)	0.957
Average number of patient consultations per week, median (Q1, Q3)	138.50 (103.75, 180.00)	125.00 (100.00, 180.00)	0.845	140.00 (100.00, 180.00)	0.385
Average number of patient consultations for mental health per week, median (Q1, Q3)	12.00 (4.95, 21.06)	4.80 (2.10, 15.00)	0.241	8.75 (2.85, 19.18)	0.439
Percentage of mental health consultations per week according to diagnosis					
Types of mental health consultation per week, median (Q1, Q3)					
Anxiety	50.00 (30.00, 70.00)	30.00 (20.00, 60.00)	0.055	50.00 (25.00, 70.00)	0.184
Depression	30.00 (20.00, 45.00)	30.00 (10.00, 50.00)	0.467	30.00 (20.00, 50.00)	0.077
Alcohol use disorders	3.00 (0.00, 10.00)	2.00 (0.00, 10.00)	0.039*	2.00 (1.00, 10.00)	0.352
Drug use disorders	2.00 (0.00, 10.00)	1.00 (0.00, 5.00)	0.037*	1.00 (0.00, 5.00)	0.553
Psychosis (including schizophrenia)	2.00 (0.00, 9.00)	2.00 (0.00, 10.00)	0.691	3.00 (0.04, 10.00)	0.074
Suicide/self-harm	1.00 (0.00, 5.00)	1.00 (0.00, 2.00)	0.254	3.00 (0.00, 3.00)	0.360
Mental health training before intervention (Jan 2015 to Jan 2016), *n* (%)					
Yes	14 (12.50)	12 (13.60)	0.486	8 (13.60)	0.976
No	98 (87.50)	76 (86.40)		51 (86.40)	
PCPs’ mental health capacities					
Knowledge about mental health, median (Q1, Q3)	6.25 (5.63, 7.50)	7.50 (6.88, 8.75)	0.684	7.50 (6.25, 8.13)	0.147
Attitudes about mental health, median (Q1, Q3)^d^	28.00 (24.00, 32.00)	25.00 (20.00, 28.00)	0.016*	27.00 (20.00, 32.00)	0.942
Self-efficacy in mental healthcare, median (Q1, Q3)	5.21 (4.08, 6.28)	7.32 (6.36, 8.05)	0.552	6.14 (5.29, 7.29)	0.796
PCPs’ referral habits, median (Q1, Q3)	50.00 (30.00, 80.00)^c^	30.00 (8.50, 60.00)	0.818	40.00 (10.00, 70.00)^c^	0.445

a. The *P*-value describes the differences in characteristics between the completers (study participants who completed the questionnaires immediately after the training) and the non-completers (study participants who did not complete the questionnaires immediately after the training) compared with pre-training. Independent t-tests for continuous variables and χ^2^ tests for categorical variables were performed.

b. The *P*-value describes the differences in characteristics between the completers (study participants who completed the questionnaires at 18 months post-training) and the non-completers (study participants who did not complete the questionnaires at 18 months after the training) compared with immediately after the training. Independent t-tests for continuous variables and χ^2^ tests for categorical variables were performed.

c. Missing values were >5% but <10%.

d. This scale is reverse scored (a higher score indicates more negative attitudes toward mental health and illness).

* *P* < 0.05.

Most PCPs included in our sample were women. At baseline, participants had a median of 49 years of age. Few PCPs reported having any mental health training in the 12 months before the implementation of the mhGAP-based training. At baseline, PCPs estimated that they saw approximately 139 patients per week, with a median of 12 patients consulting for mental health problems. Of their 36-h work week, PCPs reported dedicating <4 h to mental health. PCPs self-reported providing consultation for anxiety and depression, primarily.

Before training, PCPs scored lower on the knowledge questionnaire, higher on the attitude questionnaire, lower on the self-efficacy questionnaire and reported more referrals to specialised mental health services than immediately and 18 months after the training.^6^ No differences in characteristics were found between completers and non-completers at 18 months after training. Some differences were found between completers and non-completers immediately after training. These include scores on the attitude questionnaire (*P* = 0.016) and self-reported percentages of consultations for problems related to alcohol use (*P* = 0.039) and drug use (*P* = 0.037). However, these differences remain minimal.

The multivariable analyses presented in Table 2 shows some general trends, like statistically significant associations between PCPs’ self-reported percentage of referrals to specialised mental health services per week and several baseline characteristics across all time points. Specifically, PCPs with less experience (i.e. who were younger) reported higher levels of referrals; PCPs who reported a higher percentage of patients consulting for problems related to psychosis per week also reported higher levels of referrals; PCPs who scored higher on the mental health knowledge questionnaire reported fewer referrals to specialised mental health services.

**Table 2:** Multivariable analyses assessing the factors associated with referrals to specialised mental health services

Characteristics	Estimates	Confidence intervals	*P* value
Time			
Baseline	11.942	(1.996–21.888)	0.019*
Immediately after training	0	–	–
18 months after training	−0.179	(−9.674 to 9.315)	0.970
Group			
1	7.643	(−1.064 to 16.351)	0.084
2	0	–	–
Mental health capacities			
Knowledge about mental health	−6.330	(−9.347 to −3.314)	<0.000*
Attitudes about mental health	−0.301	(−0.967 to 0.364)	0.371
Self-efficacy in mental healthcare	−0.379	(−3.249 to 2.490)	0.793
Sociodemographic characteristics			
Age	−1.148	(−1.987 to −0.309)	0.007*
Gender			
Female	7.466	(−4.148 to 19.081)	0.205
Male	0	–	–
Practice characteristics			
Work			
Part time	6.371	(−8.836 to 21.579)	0.407
Full time	0	–	–
Previous mental health training (Jan 2015 to Jan 2016)			
No	8.774	(−4.041 to 21.589)	0.177
Yes	0	–	–
Hours of work per week	0.423	(−0.938 to 1.785)	0.538
Average number of hours dedicated to mental healthcare per week	0.020	(−0.340 to 0.381)	0.909
Average number of consultations per week	−0.026	(−0.098 to 0.046)	0.476
Average number of consultations for mental health per week	0.0889	(−0.283 to 0.461)	0.636
Percentage of consultations for anxiety per week	0.150	(−0.000 to 0.302)	0.051
Percentage of consultations for depression per week	0.081	(−0.089 to 0.253)	0.344
Percentage of consultations for alcohol use per week	0.214	(−0.172 to 0.602)	0.274
Percentage of consultations for drug use per week	−0.253	(−0.608 to 0.101)	0.160
Percentage of consultations for psychosis per week	0.385	(0.060–0.710)	0.020*
Percentage of consultations for suicide/self-harm per week	−0.084	(−0.460 to 0.291)	0.658

*P < 0.05

The multivariable analyses testing for the presence of an interaction between time and PCP study factors on referrals (Table 3) showed a significant interaction between time and attitudes about mental health on referrals to specialised mental health services per week. Higher levels of negative attitudes were significantly associated with more self-reported referrals to specialised mental health services before and at 18 months after the training programme, compared with immediately after the training. No significant interaction between time and PCPs’ knowledge and self-efficacy about mental health on referrals to specialised mental health services was found.

**Table 3:** Model controlling for all variables with time interactions for independent variables

Mental health capacities × time	Estimates	Confidence intervals	*P* value
Knowledge about mental health × time			0.156
Baseline	−4.357	(−10.190 to 1.474)	0.141
Immediately after training	0	–	–
18 months after training	1.828	(−5.596 to 9.251)	0.626
Attitudes about mental health × time			0.009*
Baseline	1.612	(0.330–2.895)	0.014*
Immediately after training	0	–	–
18 months after training	2.066	(0.5970–3.5356)	0.006*
Self-efficacy in mental healthcare × time			0.978
Baseline	0.512	(−5.055 to 6.081)	0.855
Immediately after training	0	–	–
18 months after training	−0.000	(−6.958 to 6.956)	0.999

*P < 0.05

## Discussion

This study aimed to improve understanding of the influence of PCPs’ mental health knowledge, attitudes and self-efficacy on their self-reported referrals to specialised mental health services before, immediately after and 18 months after the implementation of an mhGAP-based training. The general trend seemed to be that scores on mental health knowledge and self-efficacy questionnaires influenced PCPs’ self-reported percentage of referrals to specialised mental health services similarly across the three time points. However, we found a significant interaction between time and PCPs’ level of attitudes on referrals to specialised mental health services per week. We contextualise our findings within the literature on the theory of planned behaviour, where ‘attitudes, subjective norms and perceived behavioral control are shown to be related to appropriate sets of salient behavioral, normative, and control beliefs about the behaviour’.^19^

Our findings seem to highlight that in the backdrop of a mental health training programme, attitudes toward MNS and mental healthcare delivery may, over time, influence behaviours such as referrals to specialised mental health services more so than capacities like knowledge and self-efficacy, which we found to have no time interaction with reported referrals. Specifically, we found that higher scores on the attitude scale were associated with more referrals to specialised services before and 18 months after training, compared with immediately after training. Hence, the mhGAP training may not be enough over the longer term. Mental health stigma is still widespread in Tunisia, the Arab world and more generally in LMICs. This stigma continues to be translated into practice. For example, studies show that it is common for healthcare professionals to believe that people with mental illness are violent and dangerous, that their condition is a personal or moral fault and that treatment by mental health specialists is preferred.^16,20–24^ This reality might also be reflected in Tunisia: most consultations for mental healthcare continue to be provided in specialised services,^25^ despite the uneven distribution of mental health specialists across the country^25^ and the ministerial vision of further integrating mental health into primary care settings.^26^ Mental health stigma may be instilled in social norms and may help explain, in part, PCPs’ choice in referring to specialists versus delivering mental healthcare over the longer term despite the implementation of a mental health training.

Furthermore, the broader context consisting of public health policies and regulations may influence PCPs’ attitudes toward mental healthcare delivery and their choice in referring to specialised mental healthcare. For example, PCPs in Tunisia cannot prescribe certain medications used to treat mental health conditions;^27^ and laws before the Tunisian Revolution often considered people with problems related to substance misuse as social offenders, which may have shaped many PCPs’ ‘fear’ of these types of conditions.^27^ Organisational realities might also increase negative perceptions of mental healthcare delivery and thus referrals to specialised mental healthcare in Tunisia. For example, PCPs expressed lacking organisational support to motivate mental healthcare delivery (i.e. staff meetings to discuss challenging mental health cases, access to psychotropic medications).^27^ This larger context is often overlooked in the theory of planned behaviour. However, it might have had an effect on past experiences, which, in turn, can challenge anticipated behaviours especially over the longer term when the training's infrastructure and support may no longer be available.

Solutions to address these contextual issues should not only focus on skills-based training (like the mhGAP-based programme we offered^6^), but also on modifying policies and regulations that may help to ensure that mental health is included within universal healthcare packages and in developmental assistance plans across sectors. This inclusion may ultimately have a positive effect on mental health attitudes and thus on referrals to specialised mental healthcare. Specifically, mental health training programmes offered to non-specialists like PCPs might help to lessen their negative mental health attitudes, which can consequently effect their referrals to specialised mental health services per week immediately after the training, as shown by our current study. But, organisational supports and policies (regular supervision from specialists, the availability of psychotropic medications, changes in regulations to allow PCPs to prescribe essential psychotropic medications) are also needed to bring about a sustainable change in the attitudes of PCPs toward mental health to help maintain positive practice effects,^6,27^ including self-reported referrals. Studies also highlight the roles that healthcare organisations can play in tackling mental health stigma and in further encouraging non-specialists in mental healthcare delivery. These include strong leadership support, monitoring mechanisms related to quality of care, and opportunities for non-specialists to engage in social contact with people with lived experience who are trained in sharing their recovery journeys and experiences in navigating the healthcare system.^28–31^ These broader changes in mental health policy and legislation occurring in parallel with building non-specialists’ mental health capacities and implementing organisational supports are essential ingredients to inform mental healthcare delivery in non-specialised settings in Tunisia, and more broadly in LMICs. Interestingly, Tunisia's scale-up of a mental health training across the country, which will be offered to PCPs, will include a longer-term vision of supervision and support by specialists to further build mental health capacity in primary care settings. In addition, legislation on PCPs’ prescription of essential psychotropic medications is being revised for scale-up of the training programme. The importance of further integrating mental health into primary care settings in the country has also been acknowledged in recent reforms to the mental health curricula offered to family physicians. It now includes additional mental health courses and a mental health mandatory internship, which was previously optional.^32^ Future studies should include evaluation components related to the effect of these initiatives on attitudes toward mental healthcare delivery and their influence on referrals to specialised mental health services via primary care settings.

In LMICs, mental health specialists are already scarce, unevenly distributed and/or unavailable.^4^ Yet, they are often the ‘go to’ for mental health consultations.^4,25^ Mental health systems must be ready now and over the longer term to ensure that mental healthcare is offered to the wider population, and especially to those considered more vulnerable to the residual effects of the coronavirus 2019 pandemic.^33^ Hence, task-sharing (i.e. the increased involvement of non-specialists like PCPs in mental healthcare delivery^34–35^) may be a viable option in the context of the pandemic and beyond, should training and longer-term support be offered to non-specialists.^33^ Task-sharing will, however, continue to require changes in the conception of mental healthcare delivery of many LMICs, including Tunisia.

### Limitations and strengths

First, since our measures are based on self-reports, they could have been subject to social desirability bias, especially in the post-training measurements. Therefore, self-reports should be considered approximates. However, given that mental health statistics within clinics are not digitalised in Tunisia and thus pose a challenge in record-keeping and consultation,^27^ self-reporting was a feasible option. In addition, studies show that self-reporting practice characteristics are likely to produce reliable information in the context of measuring outcomes related to training programmes.^36^

Second, we cannot ascertain if the study's results are generalisable to all PCPs working in Tunisia. However, we assume that the influence of mental health capacities on PCPs’ self-reported percentage of referrals to specialised mental health services per week might be similar in other areas of Tunisia, should PCPs agree to participate in the training program's scale-up.

Third, two of the scales used (i.e. mental health knowledge and self-efficacy) were not previously validated. However, psychometric properties of these scales were assessed and proved acceptable.^16^

Last, we acknowledge having lost about half of our sample at 18 months post-training. However, the potential selection bias of this loss to follow-up is minimised as there was no statistical difference in the participants’ sociodemographic characteristics, mental health capacities and self-reported referrals to specialised mental health services between completers and non-completers at 18 months. In other words, study participants who remained in the study at 18 months despite a 50% attrition rate were similar to non-completers in all variables assessed (Table 1). However, some variables are worth discussing. Completers immediately after the training had statistically significant lower scores on the attitude questionnaires compared with non-completers. Had the non-completers remained in the study, level of attitudes may have been more negative and therefore may have had a greater influence on referrals to specialised mental health services. We notice that some participants (approximately 13%) had already participated in mental health training in the year before the mhGAP-based training and study enrolment. The proportion of study participants who participated in a previous mental health training remained consistent immediately and at 18 months after training. Interestingly, we observe the same trend for PCPs who had not participated in mental health training before the mhGAP-based training. For example, approximately 88% had not previously participated in a mental health training in the year before the mhGAP-based training and study enrolment, and this proportion remained at approximately 86% immediately and at 18 months after training. Hence, the level of bias related to participant engagement in mental health training is limited. It is worth noting that we lost some participants who worked part time. This finding may be explained by beliefs that mental healthcare delivery could require additional time beyond pre-existing clinical commitments.^27^ We also noticed that PCPs reported more mental health consultations per week before participating in the mental health training. We hypothesize that lower levels immediately and 18 months after training may have been because of more accurate diagnosis owing to the training and/or an overestimation of self-reported mental health consultations before training. Quality indicators for referrals in the context of training programmes like the mhGAP should be explored in future studies.

## Data Availability

The data that support the findings of this study are available from the corresponding author, J.S., upon reasonable request.
